# Impact of Virus‐Mediated Modifications in Bacterial Communities on the Accumulation of Soil Organic Carbon

**DOI:** 10.1002/advs.202506449

**Published:** 2025-05-23

**Authors:** Mingfeng Liu, Guixiang Zhou, Congzhi Zhang, Lin Chen, Donghao Ma, Lijun Zhang, Chunhua Jia, Ling Ma, Jiabao Zhang

**Affiliations:** ^1^ State Key Laboratory of Soil and Sustainable Agriculture Institute of Soil Science Chinese Academy of Sciences Nanjing 211135 China; ^2^ State Key Laboratory of Nutrient Use and Management Institute of Agricultural Resources and Environment Shandong Academy of Agricultural Sciences Jinan 250100 China; ^3^ College of Land and Environment Shenyang Agricultural University Shenyang 110866 China; ^4^ University of Chinese Academy of Sciences Beijing 100049 China

**Keywords:** auxiliary metabolic genes, carbon cycling, lifestyle, soil virus, virus‐host interactions

## Abstract

Microbial adaptations to resource availability are crucial to predict the responses of ecosystems to carbon (C) changes, yet viral roles in C cycling under varying levels of C remain elusive. Through metagenomic analysis of soils with contrasting C availability, a total of 24,789 viral contigs predominantly represent *Microviridae* and *Siphoviridae*. The soils with low C availability (straw removal) harbored 21% lysogenic viruses and enriched auxiliary metabolic genes (AMGs) related to C degradation (*p* < 0.05). Conversely, the soils with high C availability (straw returning) show 93% lytic viruses, stronger virus‐bacteria symbiosis, and numerous host functional genes related to C cycling and viral AMGs linked to C fixation (*p* < 0.05). Furthermore, these findings show that the addition of viruses boosted microbial metabolic efficiency and recalcitrant C accumulation (*p* < 0.05), with lytic activity accelerating organic C turnover via nutrient release and necromass formation. Overall, this study demonstrates viruses as key regulators of sustainable sequestration of C through host‐driven metabolic optimization.

## Graphical abstract

This study illustrates the impact of viruses on carbon cycling in soils with different availabilities of carbon. In conditions of low carbon availability, viruses adopt a lysogenic lifestyle, integrate into the host genomes and facilitate the degradation of carbon. Conversely, in high carbon availability soils, they shift to a lytic lifestyle and release nutrients that are metabolized by other microorganisms, thereby accelerating the turnover and accumulation of carbon.

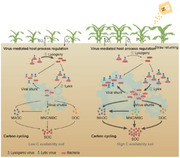

## 1 Introduction

Soil organic carbon (SOC) is the fundamental substance that maintains the stability and sustainability of terrestrial ecosystems. Soil microorganisms actively participate in the decomposition and sequestration of organic carbon (OC), which makes them indispensable for maintaining soil health and sustainable agriculture.^[^ ^1^ ^]^ While it is known that both bacterial and fungal communities are important drivers of carbon (C) cycling via “the microbial loop,” the role of viruses remains poorly understood.^[^ ^2^ ^]^ As the most abundant and diverse biological entities, viruses play a critical role in driving microbial mortality and thus, significantly impact C cycling by the release of nutrients and their effects on the microbial community composition, diversity and metabolism.^[^ ^3^, ^4^, ^5^ ^]^ As critical regulators of marine microbial loops, viral infections terminate 20–40% of bacterial biomass daily, catalytically converting ≈150 Gt C yr⁻¹ from particulate organic matter into the dissolved phase through programmed host lysis.^[^ ^6^ ^]^ These estimates are consistent with the recent 34% of fluvial C estimated to be released annually by viral lysis.^[^ ^7^ ^]^ While the importance of viruses for C cycling in the oceans is well established, the association of viruses with the turnover of C and the sequestration of OC in soils remains elusive.^[^ ^6^, ^8^, ^9^ ^]^

When examining the potential implications of viruses in the regulation of soil C cycling, two categories have been considered, including top‐down and bottom‐up regulatory mechanisms.^[^ ^6^, ^10^ ^]^ The top‐down regulatory mechanism primarily involves that viruses lyse hosts, altering functional microbe abundance and community structure. The intracellular compounds released by cell lysis can then directly enter metabolic pathways, reshaping the abundance, diversity, and community structure of microorganisms.^[^ ^4^, ^11^, ^12^, ^13^ ^]^ Conversely, the bottom‐up regulatory mechanism involves viruses in lysogeny where viruses maintain a latent state within their hosts.^[^ ^14^ ^]^ Viruses can harbor auxiliary metabolic genes (AMGs), which are derived from their hosts. When these AMGs are introduced into new hosts via horizontal gene transfer (HGT), they may modulate microbial metabolic pathways, indirectly regulating the C cycling in terrestrial ecosystems.^[^ ^13^, ^15^ ^]^ Over the last decade, viral AMGs linked to C cycling have been identified. Most previous studies have shown that the potential AMGs can modify the metabolic functions of bacteria and thus, indirectly regulate the processes of C cycling.^[^ ^8^, ^16^ ^]^ Emerging evidence reveals that AMGs critically mediate microbial survival strategies under C‐limited conditions. When facing C scarcity, soil microorganisms activate metabolic pathways to synthesize diverse extracellular enzymes, enabling depolymerization of recalcitrant substrates like chitin and lignin‐derived compounds.^[^ ^17^ ^]^ To date, the studies of soil viruses have focused on exploiting previously sequenced metagenomic datasets. However, current evidence remains indirect, relying on inferred microbial population shifts and AMG presence.^[^ ^8^, ^13^, ^16^ ^]^ While microcosm experiments confirm viral top‐down effects, the absence of microbial sequencing data limits holistic assessments of viral impacts on C cycling.^[^ ^3^, ^9^ ^]^

In China's North China Plain, that is, a critical grain production region, straw returning and nitrogen (N) fertilization are key strategies for enhancing agricultural soil C sequestration.^[^ ^18^, ^19^ ^]^ Yet, we still have a limited understanding on how viruses and bacteria jointly drive C cycling under these practices remains unclear. In light of the extensive research needed to understand these factors, our aim was to address this gap. For this purpose, our study focused on two goals: 1) elucidating how viral and bacterial communities respond to varying C availability under straw returning and N application, and 2) linking these responses to SOC accumulation in typical fluvo‐aquic soils. Specifically, we sought to quantify SOC components: microbial necromass C (MNC), microbial biomass C (MBC), mineral‐associated OC (MAOC), particulate OC (POC), dissolved OC (DOC), Fe‐bound OC (Fe‐OC), and Ca‐bound OC (Ca‐OC). We then Analyzed microbial communities, viral lifestyles, and AMGs via meta‐viromic and metagenomic approaches. Moreover, the microcosmic experiments were conducted to visually demonstrate how viruses regulate their host communities to drive the accumulation of SOC. This work establishes viruses as critical agents in soil C cycling, providing insights for sustainable agriculture.

## 2 Results

### 2.1 Soil Organic Carbon and its Components

The long‐term experimental results showed that straw returning and N application significantly enhanced SOC and its components (Table S2, Supporting Information). Specifically, with the straw returning and N application, the contents of SOC and its components, that is, microbial‐derived C, POC, and MAOC showed marked increases (*p* < 0.05). Compared with T1, the content of MBC in T2, T3, and T4 increased by 288% (T2), 127% (T3), and 569% (T4), while MNC increased by 33%, 36%, and 77% across these treatments. Furthermore, the contents of MAOC and POC increased significantly after straw returning and N application. These observations were confirmed by reactive mineral‐phase C measurements, that is, Ca‐OC and Fe‐OC. Among them, the highest increase in the components of SOC was observed in T4, thus, directly illustrating that straw returning combined with the N application treatment facilitated the sequestration of C in agricultural soil.

### 2.2 Relationship between the Viral and Bacterial Communities and the SOC Components

**Figure 1:**
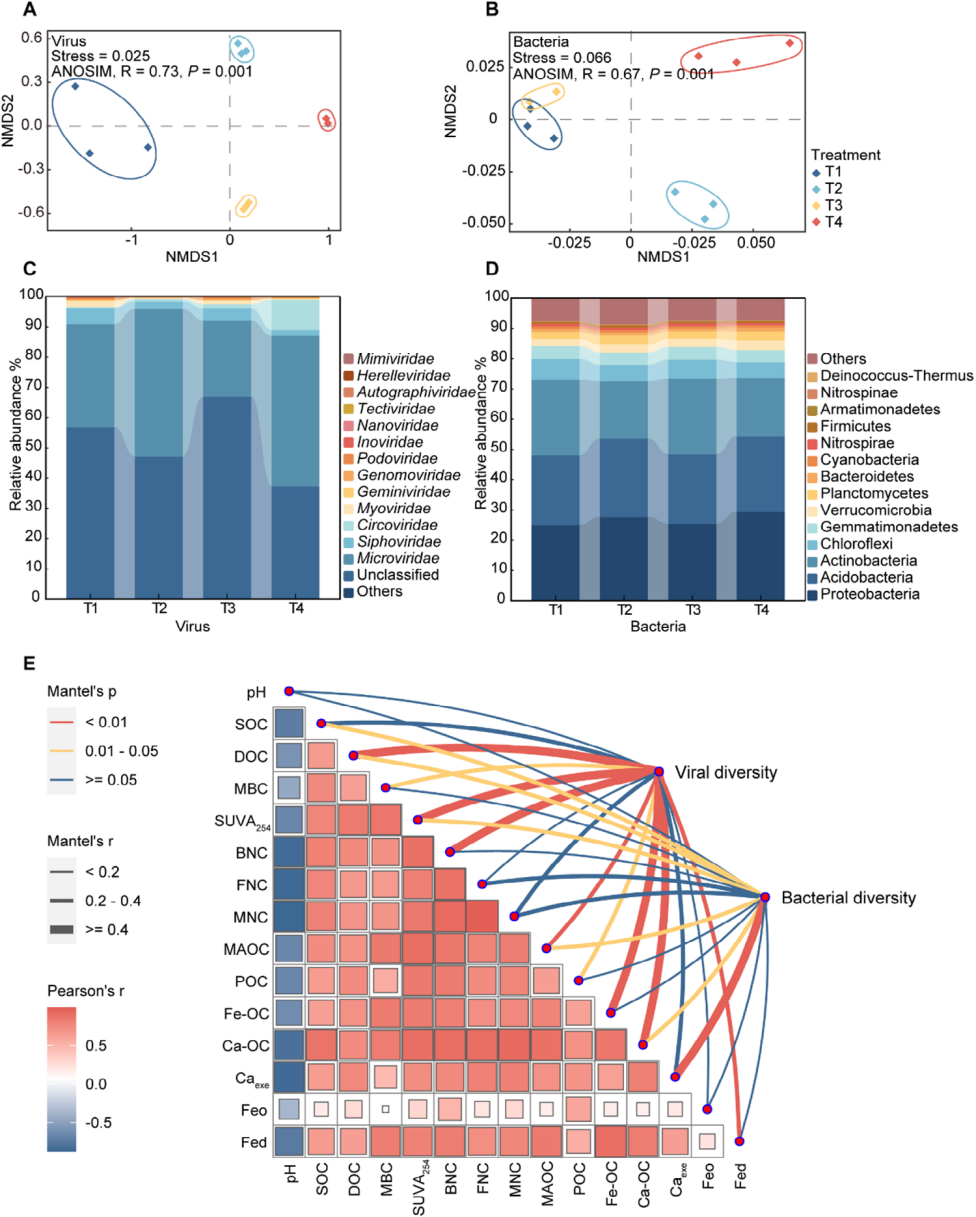
Diversity and composition of the viral and bacterial communities in the soils and potential factors that contribute to the variation in viral and bacterial diversity. NMDS analysis of the A) viral and B) bacterial communities using Bray‐Curtis similarities. The significance was assessed via an ANOSIM. The taxonomic composition of C) viral and D) bacterial contigs identified from four treatments, with viral classification at the family level and bacterial classification at the phylum level. E) The Mantel test between the first dimension (NMDS1) of the viral and bacterial communities and SOC components. ANOSIM, analysis of similarity; NMDS, Non‐metric Multi‐Dimensional Scaling; SOC, soil organic carbon; DOC, dissolved organic carbon; MBC, microbial biomass carbon; SUVA_254_, a typical DOC aromaticity index; BNC, bacterial necromass carbon; FNC, fungal necromass carbon; MNC, microbial necromass carbon; MAOC, mineral‐associated organic carbon; POC, particulate organic carbon; Fe‐OC, C associated with the reactive Fe mineral phases; Ca‐OC, C associated with the cations; Ca_exe_, exchangeable Ca; Feo, oxalate‐extractable Fe; Fed, dithionite‐extractable Fe; T1, straw removal without N fertilization; T2, straw returning without N fertilization; T3, straw removal with N fertilization; T4, straw returning with N fertilization.

To explore linkages between C turnover and microbial dynamics, we analyzed the diversity of the bacterial and viral communities via the use of metagenomic and meta‐viromic contigs. Across all soil treatments, 24 789 viral contigs (> 5 kb) were identified, with dominant families including ssDNA viruses (*Microviridae*, *Geminiviridae*) and dsDNA viruses (*Siphoviridae*, *Myoviridae*) (**Figures** **1**A,C, and S1A, Supporting Information). In particular, *Microviridae* abundance significantly increased in the straw returning soils (T2 and T4) (*p* < 0.05). The relative abundance of the ssDNA viruses *Circoviridae* increased from 0.32% (T1) to 9.88% (T4) (Figure 1C and Table S3, Supporting Information). Additionally, both the alpha‐diversity and beta‐diversity of the viral communities were clearly changed by straw returning and N application (Figure 1A and Figure S1A, Supporting Information). The annotated bacterial phyla were predominantly Proteobacteria, Acidobacteria, and Actinobacteria (Figure 1D). Compared to T1, Straw returning significantly increased relative abundance of Proteobacteria and Acidobacteria (*p* < 0.05) and reshaped bacterial beta‐diversity, though alpha‐diversity remained stable (Figure 1B and Figure S1A, Supporting Information).

The Mantel tests showed strong associations between microbial communities and SOC components (Figure 1E and Figure S1B, Supporting Information). Some notable positive correlations were observed between the contents of SOC components (*p* < 0.01). We found that soil DOC, SUVA_254_ (a typical DOC aromaticity index), MBC, bacterial necromass carbon (BNC), dithionite‐extractable Fe (Fed), Ca‐OC, Fe‐OC and MAOC were significantly associated with the viral diversity (*p* < 0.05, Figure 1E). While viral community composition was primarily driven by SOC and POC (*p* < 0.05, Figure S1B, Supporting Information). Additionally, significant correlations were observed between the bacterial diversity and SOC, DOC, SUVA_254_, exchangeable Ca (Ca_exe_), Ca‐OC and MAOC (*p* < 0.05). These findings suggest that the SOC components were drivers of the dissimilarity in the viral communities.

### 2.3 Lifestyle of the Viral Communities

**Figure 2:**
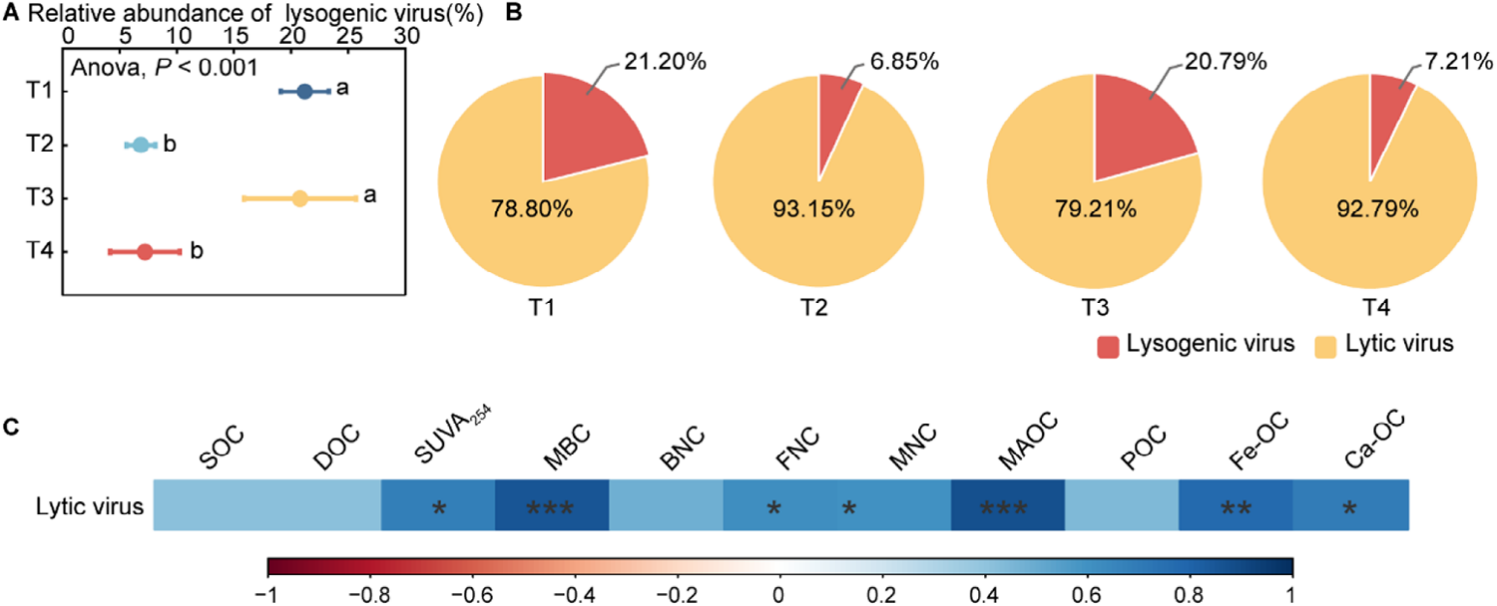
Comparison of the viral lifestyles and the correlation between lytic viruses and SOC components. The relative proportions of the lytic and lysogenic viruses across the different treatments A,B), with significant differences denoted by lowercase letters (ANOVA, *p* < 0.05). C) A correlation analysis depicting the relationship between the relative abundance of lytic viruses and SOC components. SOC, soil organic carbon; DOC, dissolved organic carbon; MBC, microbial biomass carbon; SUVA_254_, a typical DOC aromaticity index; BNC, bacterial necromass carbon; FNC, fungal necromass carbon; MNC, microbial necromass carbon; MAOC, mineral‐associated organic carbon; POC, particulate organic carbon; Fe‐OC, C associated with the reactive Fe mineral phases; Ca‐OC, C associated with the cations; T1, straw removal without N fertilization; T2, straw returning without N fertilization; T3, straw removal with N fertilization; T4, straw returning with N fertilization. *p < 0.05. **p < 0.01. ***p < 0.001.

The lysogenic marker, including integrase, transposase, invertase, and combined proteins, within the viral contigs were identified to assess the proportion of free lysogenic viruses. In total, 8106 viral contigs were identified as lysogenic viruses, while the remaining contigs without genes specific to lysogeny or prophage signals were inferred as potentially lytic viruses. As expected, lytic viruses dominated fluvo‐aquic soils, constituting 78.8%–93.2% of total viral communities (**Figure** **2**B). Moreover, the straw returning treatments (T2 and T4) exhibited significantly lower lysogenic virus abundance compared to the straw removal treatments (T1 and T3, Figure 2A,B). Furthermore, although both lysogenic and lytic communities were primarily composed of dsDNA viruses (*Siphoviridae*, *Myoviridae*). The lytic viruses displayed greater taxonomic diversity, with *Microviridae* emerging as the most abundant lytic family (Figure S2, Supporting Information). In addition to these findings, our results revealed that the relative abundance of lytic viruses strongly positively correlated with the MBC, MNC, MAOC and SUVA_254_ (*p* < 0.05, Figure 2C). This clearly showed that not only demonstrate viral community linkages to SOC components but also suggest viral lifestyle as a potential driver of C sequestration.

### 2.4 Potential Virus‐Host Interactions

**Figure 3:**
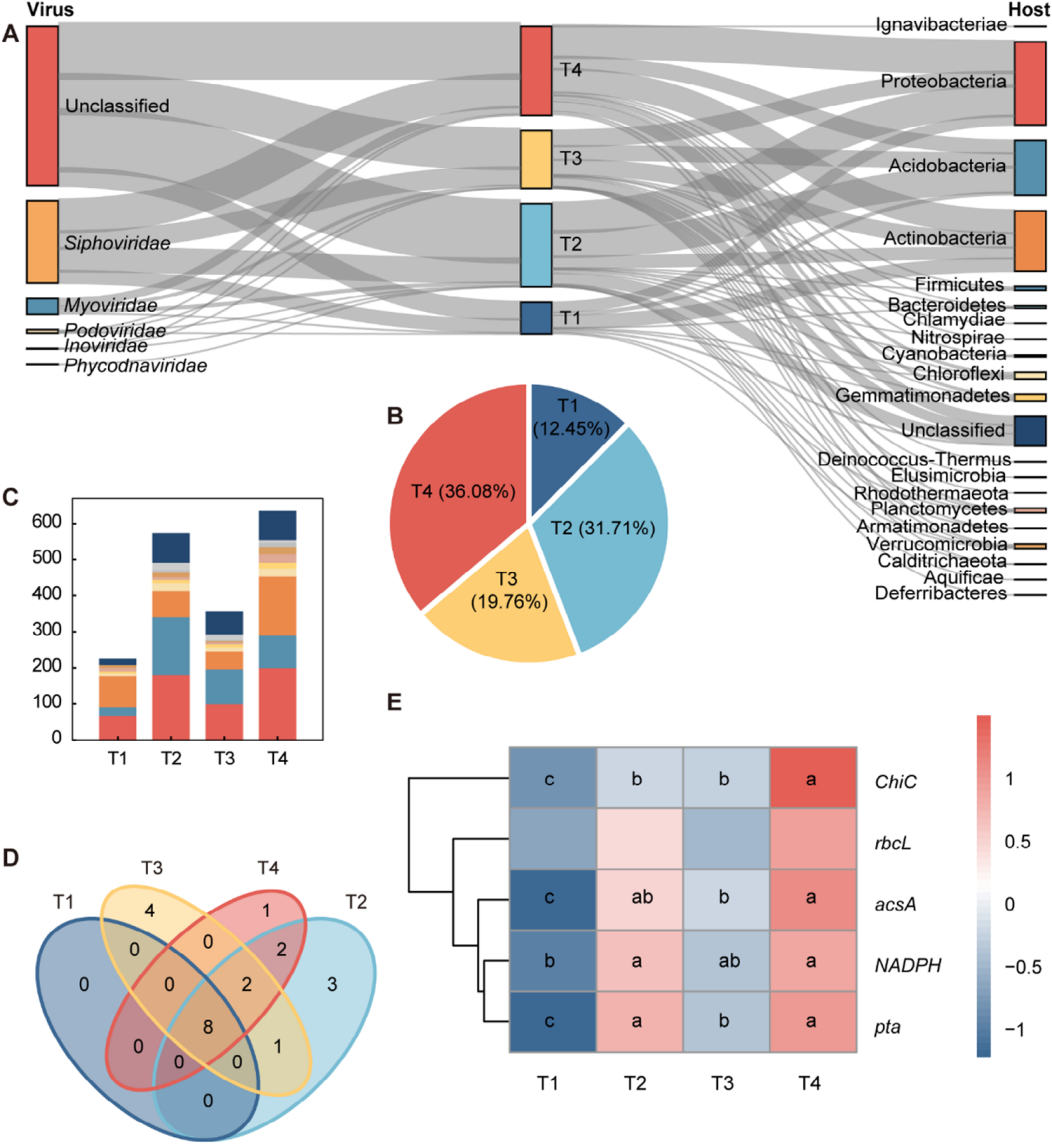
Analysis of the virus‐host links and associated host functions. A) Predicted links between the viral taxonomy (at the family level) and bacterial taxonomy (at the phylum level). The middle column illustrates the frequency of virus‐host links. B) Pie chart showing the proportion of the virus‐host links across treatments. C) Number of the predicted hosts at the phylum level under different treatments. Column colors correspond to the host taxonomy color scheme in right of (A). D) Venn diagram of the shared and unique host phyla among all treatments. E) The abundance of functional genes related to C cycling harbored by the host, with abundance normalized across treatments based on the Z‐score and significant differences denoted by lowercase letters (ANOVA, *p* < 0.05). T1, straw removal without N fertilization; T2, straw returning without N fertilization; T3, straw removal with N fertilization; T4, straw returning with N fertilization.

To elucidate virus‐host interactions and their ecological implications, we predicted 1807 virus‐host links spanning 409 viral contigs and 824 host contigs (**Figure** **3**A and Table S8, Supporting Information). Among these interactions, 133 viral contigs were connected with a single host, while the remaining viruses had a broad host range and were linked to bacteria from several bacterial phyla. These viruses were primarily classified as *Siphoviridae* and *Myoviridae*. Most of the viral hosts detected were assigned to the Proteobacteria, Acidobacteria, and Actinobacteria phyla, which also represent the dominant soil microorganisms (Figure 1D and Figure 3A, Supporting Information). The straw returning treatments (T2 and T4) were observed to have significantly more virus‐host links compared to the straw removal treatments (T1 and T3, Figure 3B) with enriched host diversity in amended soils including Actinobacteria, Firmicutes and Nitrospirae (Figure 3A,C,D). Functional annotation of hosts revealed enhanced C metabolic capacity under straw returning. Notably, hosts showed elevated abundances of C cycling genes compared to T1, including *ChiC* associated to amino sugar metabolism (*p* < 0.001), *acsA* (*p* < 0.01), *pta* (*p* < 0.001) and *NADPH* (*p* < 0.05) related to C fixation pathway (Figure 3E and Table S12, Supporting Information).

### 2.5 Viral AMGs

**Figure 4:**
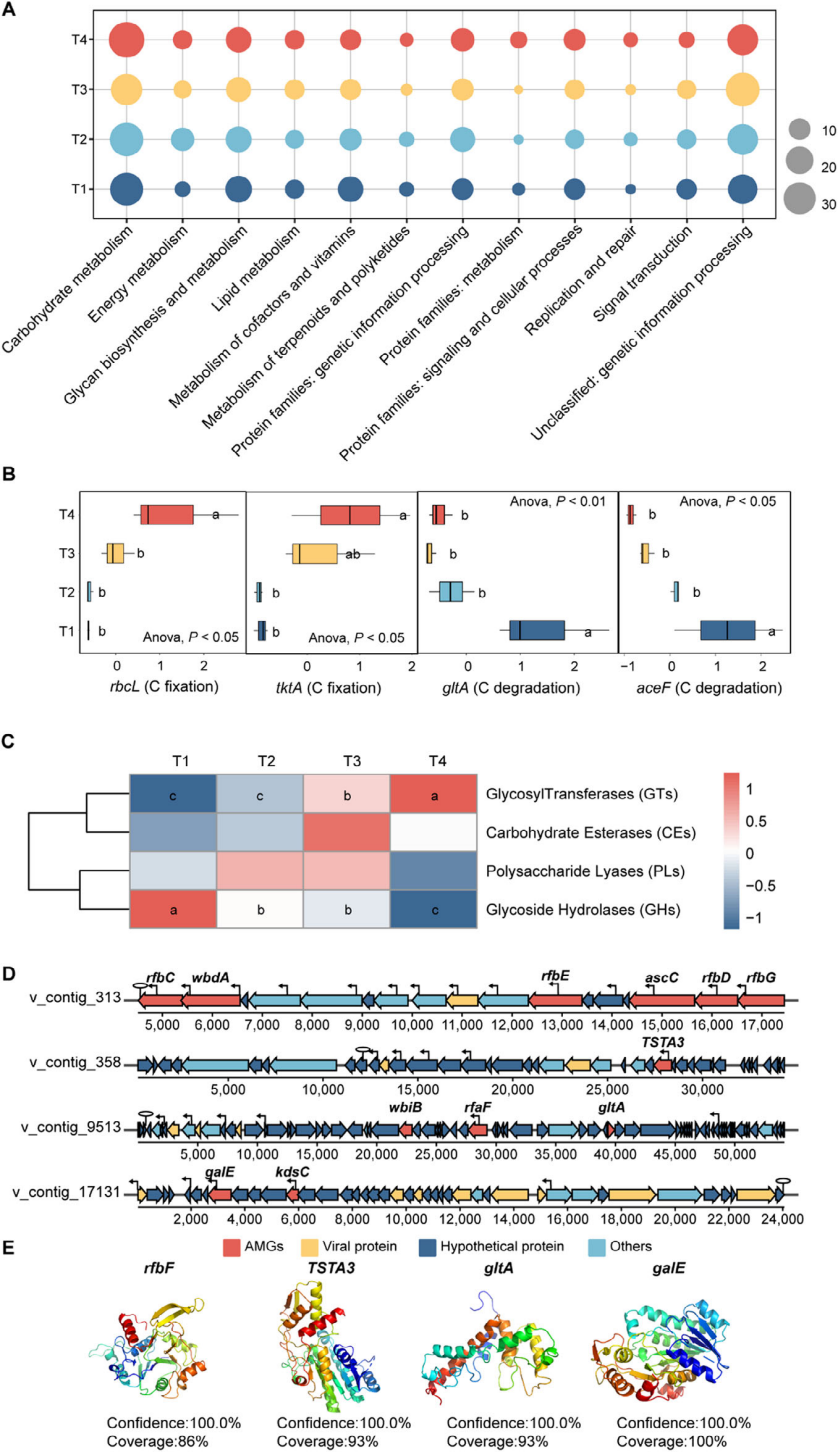
Auxiliary metabolic genes (AMGs) encoded by viruses. A) Abundance of viral AMGs annotated in the KEGG database (level 2). B) The box plots show the abundance of AMGs related to C fixation and C degradation, with abundance values normalized among the different treatments using a Z‐score and significant differences denoted by lowercase letters (ANOVA, *p* < 0.05). C) Heatmap shows the abundance of AMGs in the CAZymes database, with abundance values normalized among the different treatments using a Z‐score and significant differences denoted by lowercase letters (ANOVA, *p* < 0.05). D) Genetic map of the AMGs. Arrow color, gene types; arrow length, size of the open reading frames (ORFs). E) Tertiary structures of the viral proteins expressed by the viral AMGs involved in C cycling. AMGs, auxiliary metabolic genes; KEGG, Kyoto Encyclopedia of Genes and Genomes; CAZymes, carbohydrate‐ active enzymes; T1, straw removal without N fertilization; T2, straw returning without N fertilization; T3, straw removal with N fertilization; T4, straw returning with N fertilization.

**Figure 5:**
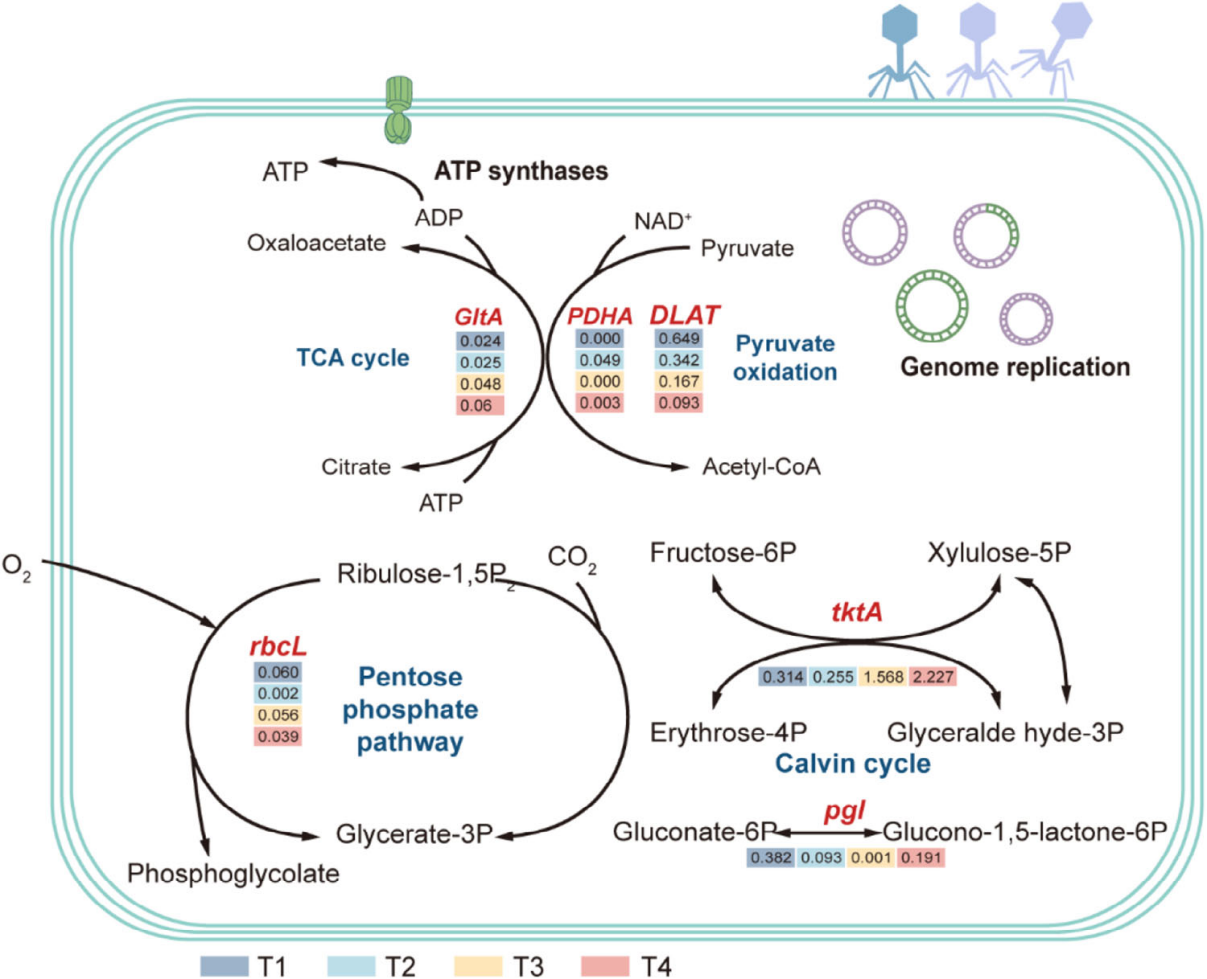
Conceptual diagrams that depict the virus‑host interactions via AMGs. The diagram shows how viruses may modulate C metabolism of the hosts. Red fonts, AMGs; AMGs, auxiliary metabolic genes; color squares below the AMGs, relative abundance; T1, straw removal without N fertilization; T2, straw returning without N fertilization; T3, straw removal with N fertilization; T4, straw returning with N fertilization.

To obtain a more comprehensive understanding of viral role in soil C cycling, we identified a total of 4403 AMGs associated with 264 Kyoto Encyclopedia of Genes and Genomes (KEGG) homologs (KOs) using VIBRANT and DRAMV analyses (Table S15, Supporting Information). They revealed enrichment of the metabolic AMGs involved in carbohydrate metabolism, glycan biosynthesis and metabolism, cofactors and vitamins metabolism, and energy metabolism, while the non‐metabolic AMGs primarily involved genetic information processing (**Figure** **4**A). Notably, the abundance of AMGs associated with C cycling varied across the different treatments. In particular, the AMGs associated with C fixation were the most frequent and active in T4, including *rbcL* (*p* < 0.05) and *tktA* (*p* < 0.05). The AMGs related to C degradation were more abundant in T1, including *gltA* (*p* < 0.01) and *aceF* (*p* < 0.05, Figures 4B and **5**). Similar results were obtained using the dataset of carbohydrate‐active enzymes (CAZymes). A total of 39 AMGs annotated as CAZymes across four functional classes, including polysaccharide lyases (PLs), glycosyltransferases (GTs), carbohydrate esterases (CEs) and glycoside hydrolases (GHs, Figure 4C). In detail, the viruses in T1 encoded a higher proportion of AMGs related to GHs (*p* < 0.01), whereas those in T3 and T4 had significantly enriched AMGs related to GTs, which promote the formation of polysaccharides (*p* < 0.05). Moreover, putative AMGs were detected on five completely closed viral genomes. By scrutinizing the upstream and downstream genomes of the AMGs and comparing common viral genes, such as viral structural genes, promoters, and terminators, we identified genomic backgrounds with high confidence. They encompass various functional genes related to the C cycling (Figure 4D). Furthermore, structural modeling via Phyre2 predicted 100% confidence in viral AMG functionality, supporting their potential expression (Figure 4E). These results confirmed that the viruses had important potential roles in the soil C dynamics through the expression of putative AMGs related to C cycling within the host genomes.

### 2.6 The Impact of the Addition of Viruses on SOC

**Figure 6:**
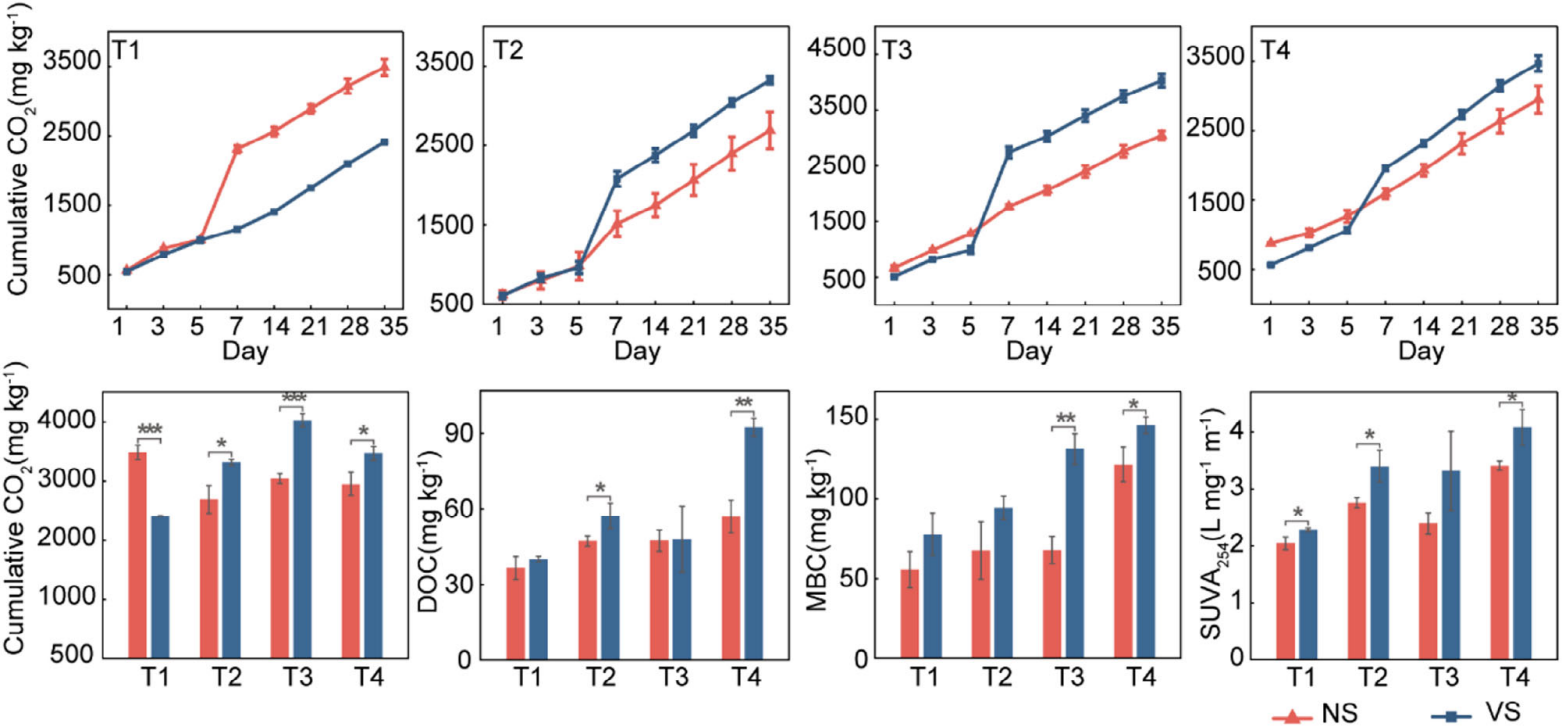
Impact of the addition of viruses on the C cycling components. CO_2_, carbon dioxide; DOC, dissolved organic carbon; MBC, microbial biomass carbon; SUVA_254_, a typical DOC aromaticity index; NS, non‐addition of virus treatment; VS, addition of virus treatment; T1, straw removal without N fertilization; T2, straw returning without N fertilization; T3, straw removal with N fertilization; T4, straw returning with N fertilization. Significant differences were based on a Student's *t*‐test. *p < 0.05. **p < 0.01. ***p < 0.001.

We conducted a microcosm experiment by adding viral concentrates to test their effects on soil microbial communities and OC (Figure S3, Supporting Information). The carbon dioxide (CO_2_) emissions without the addition of viruses exceeded those with viruses in T1 within 7 days of incubation. However, this pattern reversed in other treatments (T2, T3, T4). After 7 days, the CO_2_ emissions tended to stabilize across all groups without significant differences. By the end of incubation, the cumulative production of CO_2_ in T1 ranked NS (non‐addition of virus treatment) > VS (addition of virus treatment, *p* < 0.05), while T2, T3, and T4 showed VS > NS (*p* < 0.05, **Figure** **6** and Table S20, Supporting Information). Furthermore, we also assessed the other components of C cycling in our system and observed viral impacts. Generally, viral addition increased the levels of DOC, SUVA_254_, and MBC, particularly in T4 (*p* < 0.05, Figure 6). Moreover, the microbial metabolic quotient (respiration per unit microbial biomass, qCO_2_) was used to quantify the metabolic efficiency to illustrate the influence of soil viruses. The addition of viruses significantly reduced the qCO_2_ of the soils with straw removal (T1 and T3), but had no observable differences in the straw returning soils (T2 and T4, Figure S4C, Supporting Information). Additionally, the addition of viruses stimulated the activities of the soil enzymes related to the degradation of sugar and chitin, including β‐glucosidase (BG) and N‐acetyl‐glucosidase (NAG, Figure S4A,B, Supporting Information). In addition, a random forest analysis indicated that SUVA_254_, DOC, BG and NAG as key drivers of qCO_2_ (*p* < 0.05, Figure S4D, Supporting Information). Overall, these results demonstrated that the addition of viruses may alter microbial growth dynamics, stimulate the activities of enzymes, and thus, facilitate the accumulation of DOC.

## 3 Discussion

### 3.1 Characteristics of the SOC Components

The availability of C plays a pivotal role in shaping the composition, diversity, and interactions among microorganisms by influencing the ecosystem processes and stability.^[^ ^20^ ^]^ Our results revealed that the long‐term straw returning treatments (T2 and T4) resulted in a significantly higher C availability and accumulation of the SOC components, such as MNC, MBC, MAOC, and POC (Table S2, Supporting Information). Conversely, significantly less C was available in the soils subjected to the long‐term applications of fertilizer with straw removal (T1 and T3). Despite the growing recognition of the interplay between the availability of soil C and microorganisms, our understanding of how the availability of C drives the allocation of microbial nutrients and profoundly influences the ecological functions of the microbial community remains preliminary.^[^ ^21^ ^]^ Thus, to facilitate a clearer discussion, we used the soil with low C availability to represent T1 and T3, and the soil in which the C was highly available to represent T2 and T4 (Table S1, Supporting Information).

We found positive associations among the SOC components (Figure 1E). Recent studies elucidated that under the conditions of limited nutrients, the microbial communities prioritize their resources to sustain their growth, which results in the preferential breakdown of the microbial necromass by soil microorganisms using extracellular enzymes to fulfill the requirements for C and nutrients.^[^ ^22^, ^23^, ^24^ ^]^ We concluded that soil with low C availability could stimulate decomposition by microorganisms, which led to the loss of C. In comparison, agricultural management drives the microbial communities to allocate resources in favor of the formation of microbial necromass, thereby accelerating the turnover of microorganisms.^[^ ^25^, ^26^, ^27^ ^]^ Consequently, straw returning soils with high C availability could lead to the accumulation of microbial‐derived C, which subsequently enhances the mineral protection of SOC from enzymes and microbial decomposition and elevates the proportion of complex components within the pool of DOC.^[^ ^5^ ^]^

### 3.2 The Influence of Viral Community on the Accumulation of SOC

In this study, we examined the role of viruses in C cycling that varied in their availability of C by utilizing multi‐omics techniques. The findings of this study were consistent with those of the previous soil studies, and the ssDNA viruses *Microviridae* and *Circoviridae* and the dsDNA *Siphoviridae* were identified as the predominant populations of viruses.^[^ ^16^, ^28^ ^]^ Since we used the multiple displacement amplification method, which preferentially amplifies ssDNA, there were high levels of ssDNA viruses.^[^ ^29^, ^30^, ^31^, ^32^ ^]^ Additionally, the viral communities changed closely with the different C availability. In contrast, there were no significant differences in the alpha‐diversity of the bacterial communities (Figure 1A and Figure S1A, Supporting Information). Numerous research highlights that the diversity of bacterial communities demonstrates a more pronounced response to the application of phosphorus (P) fertilizer.^[^ ^33^, ^34^, ^35^, ^36^ ^]^ In comparison, the soils in this study that were treated with the same amount of P could lead to bacterial communities with similar levels of diversity. Moreover, the C derived from microorganisms and the OC associated with minerals had significant relationships with the viral community, particularly in terms of diversity (Figure 1E and Figure S1C, Supporting Information). Together, these results indicate that the viral communities were coupled with the SOC components.

Furthermore, the lifestyles of soil viruses profoundly impacted the survival status of the viral communities. Our study elucidated that viruses in all the samples predominantly adopt a lytic lifestyle (Figure 2A,B), which indicated that the viral communities in the fluvo‐aquic soils primarily influence the microbial communities by lysing their hosts. In this study, we found that the soils with low C availability had a higher abundance of lysogenic viruses in comparison to the soils with high C availability, which indicated that the viruses tend to adopt a lysogenic lifestyle in the environments with limited amounts of C (Figure 2A). This result showed that soil viruses in a dormant state, including integrated prophages and pseudolytic genomes, remain inactive during the infection of host cells. This state may temporarily halt their activity under harsh environmental conditions, but viruses can enhance their adaptability, which enhances their survival, and reinitiate their replication cycle when environmental conditions improve.^[^ ^37^, ^38^, ^39^, ^40^ ^]^ In comparison, the proportion of lytic viruses in soils that had high C available reached as high as 93%, which showed that the viruses in the soils rich in C tend to influence microbial growth that is in a lytic state. In addition, the community composition of the lytic viruses was rich in ssDNA viruses, which could potentially lead to frequent mutational events and a more diverse community composition.^[^ ^41^ ^]^

Similarly, we attempted to explore the contribution of the viral life cycle to soil C cycling. The positive correlation between the abundance of lytic viruses and the content of C components suggested that lytic viruses significantly promote OC accumulation (Figure 2C). This may occur through two key mechanisms: nutrient release, viral lysis releases intracellular nutrients and enzymes into the soil; niche vacancy, host cell death creates ecological niches for other microorganisms, which is consistent with “viral shunt” process.^[^ ^30^ ^]^ These processes stimulate microbial metabolic activity, increasing microbial‐derived C (e.g., MAOC stabilized by minerals and complex DOC). Notably, viral activity remobilizes intracellular C faster than N or P, potentially reducing C loss.^[^ ^42^ ^]^ Collectively, lytic viruses play a pivotal role in C sequestration, particularly in soils with high C availability. Supporting this, studies show that viral‐driven microbial turnover contributes 33%–62% of stabilized SOC in temperate systems, highlighting viruses as key regulators of soil C cycling.^[^ ^43^, ^44^ ^]^

### 3.3 The Impact of Virus and Host Functional Composition on Carbon Cycling

Characterization of bacterial communities showed that viruses could also profoundly impact the diversity and composition of these communities (Figures 1 and 3). To clarify viral roles in shaping microbial metabolism under varying C availability, we linked viral contigs to bacterial hosts. This study revealed a large number of generalist viruses, such as *Siphoviridae* and *Myoviridae*, that infect the dominant bacterial phyla. The viruses had traits of varied efficiencies of infection, patterns of abundance, and other distinctive properties (Figure 3A).^[^ ^45^ ^]^ Furthermore, in high C availability soils, host prediction revealed viruses preferentially infected dominant copiotrophs (e.g., Bacteroidetes, Proteobacteria), which generally exhibit elevated metabolic rates and rapid growth. Infected Firmicutes, known for fermentation genes (e.g., ethanol/lactate production), may reflect viral preference for metabolically active hosts.^[^ ^45^ ^]^ These results are consistent with “Kill the Winner” hypothesis, which suggests that viruses selectively target the dominant group of host bacteria, thus, reducing their dominance effects and evening out the competition between different taxa of bacteria.^[^ ^46^, ^47^ ^]^ A network analysis revealed that the communities of both viruses and hosts in the soils with high C availability promote the complexity and robustness of networks (Figure S5, Supporting Information). Additionally, the hosts harbor an increasing abundance of the functional genes associated with C cycling (e.g., *ChiC*, *acsA*, *NADPH*, and *pta*, Figure 3E). Taken together, these findings highlight that in soils with high C availability, frequent virus‐host interactions drive dominant bacteria turnover. Released organic matter and vacated niches support metabolic diversification, ultimately enhancing microbial functional capacity for C processing, enhancing the complexity and robustness of the host network.

Numerous studies have highlighted the ability of viruses to acquire auxiliary metabolic genes (AMGs) through horizontal gene transfer (HGT) during infection, enabling them to reprogram host metabolism and influence biogeochemical cycles across diverse environments.^[^ ^11^, ^48^ ^]^ In this study, we identified a substantial diversity of AMGs linked to C metabolism, further confirming their pivotal role in soil C cycling (Figure 4). In particular, we noted a higher abundance of AMGs related to glycoside hydrolases and C degradation (e.g., *gltA, aceF*) in soils with low C availability. These carbohydrate‐active AMGs likely enhance host capacity to break down complex polysaccharides, as observed in nutrient‐limited mangrove sediments.^[^ ^48^ ^]^ By facilitating microbial C degradation, these AMGs carried by soil viruses may improve host fitness by expanding access to resources in resource‐scarce environments.^[^ ^11^, ^15^, ^17^, ^49^ ^]^ Consequently, the AMGs linked to glycoside hydrolases identified in the conditions with low C availability may enhance the abilities of the bacteria to degrade C, which supports the growth and reproduction of viruses and their hosts. In contrast, Viruses preferentially harbored AMGs linked to C fixation (e.g., *rbcL, tktA*) and glycosyltransferases (GTs) under conditions with high C availability (Figures 4B,C, and 5). These AMGs associated with the pentose phosphate pathway and the Calvin cycle share bidirectional enzymes. Calvin cycle AMGs utilize reducing power to fix inorganic C, while pentose phosphate pathway AMGs boost host deoxyribonucleotide triphosphates (dNTP) synthesis, aiding viral replication.^[^ ^42^, ^50^ ^]^ Additionally, AMGs related to GTs promote structural polymer biosynthesis (e.g., cellulose), directly contributing to OC accumulation.^[^ ^48^ ^]^ Collectively, these findings suggest that viral AMGs strategically optimize host metabolism under varying C conditions, that is, enhancing decomposition in soils with low C availability and favoring SOC storage in high C availability aligns with the ecological strategies of oligotrophs and copiotrophs, respectively, mirroring their divergent resource acquisition priorities.^[^ ^51^ ^]^ This dual role underscores viruses as metabolic “tuners” in soil C dynamics.

### 3.4 Virus Additions Impact the Accumulation of Carbon

To directly assess viral impacts on soil C dynamics, we conducted a microcosm experiment tracking CO₂ emissions and DOC changes. Generally, CO₂ release followed a first‐order kinetic pattern: rapid initial microbial growth under abundant resources, then stabilization as carrying capacity was reached.^[^ ^9^ ^]^ In particular, the initial rapid growth of microorganisms was observed owing to sufficient space and resources. However, as the system approached carrying capacity, the microbial growth stabilized with diminishing spatial resources. In soils with low C availability and a low biomass and initial nutrient content, the addition of viruses may lead the hosts to lyse. This results in a reduction in overall biomass and metabolic activity, thus, decreasing respiration rates (Figure S4C, Supporting Information). Conversely, in soils with high C availability and richer concentrations of nutrients, the addition of viruses would likely lower bacterial populations and CO₂ emissions but enhanced surviving microbial metabolism through two pathways. On the one hand, lysed cells liberated intracellular organic matter, expanding niche space. On the other hand, elevated enzyme activity increased microbial respiration efficiency (Figure S4C, Supporting Information). Notably, the soils that had been supplemented with viruses significantly increased the content and complexity of DOC in soils with high C availability, favoring its conversion to stable OC (Figure 6 and Figure S5, Supporting Information).^[^ ^9^, ^52^ ^]^ Moreover, the random forest analysis highlighted the improvement in the microbial C metabolic efficiency following the addition of viruses that could possibly be related to the presence of recalcitrant DOC and the activity of enzymes (Figure S4D, Supporting Information). These effects confirm the previous metagenomic conclusion and highlight viruses as modulators of soil C fate.

**Figure 7:**
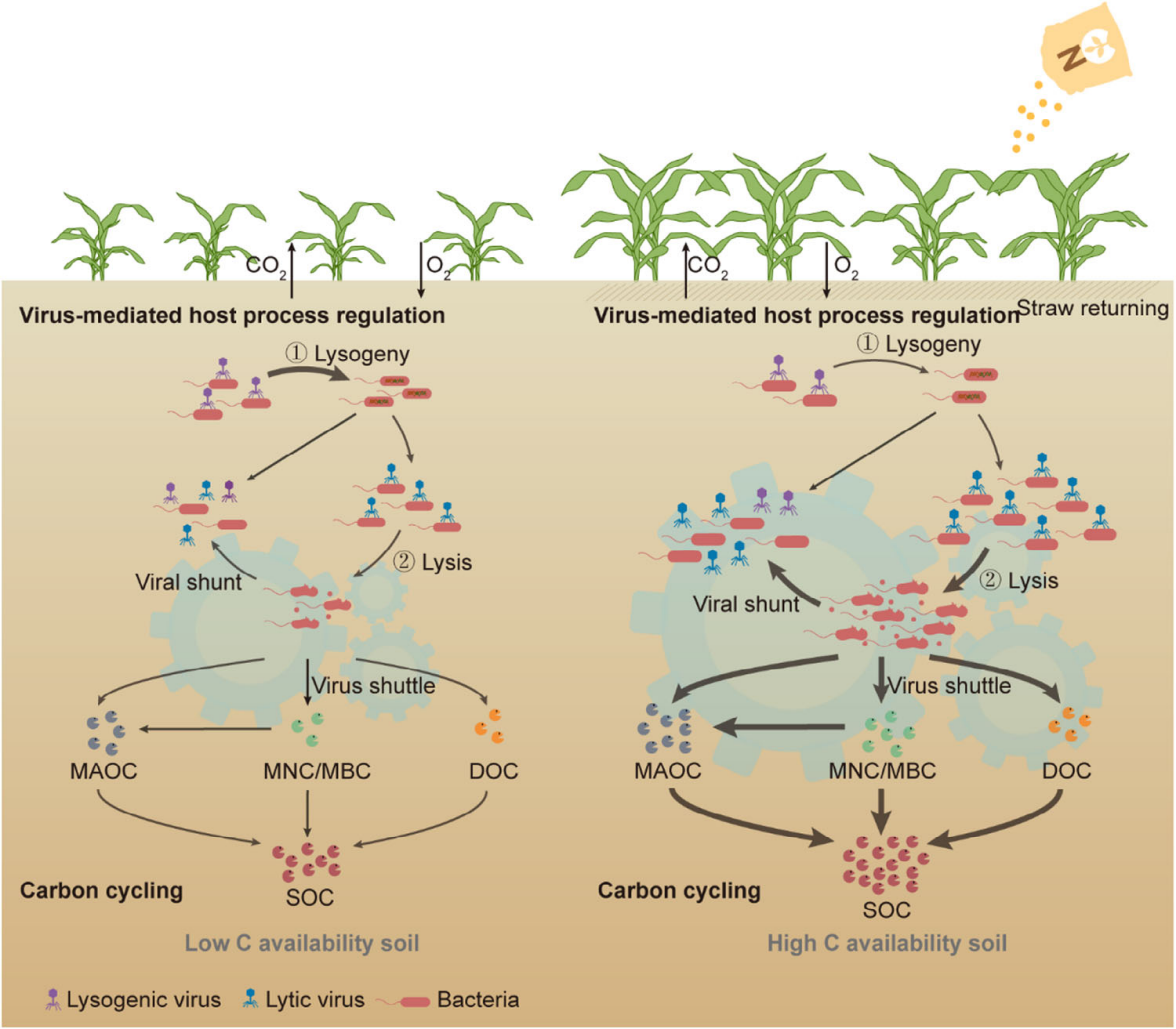
Viral regulation of soil carbon (C) cycling under long‐term straw returning and N fertilization. Conceptual model illustrating virus‐mediated C dynamics in contrasting C availability regimes: 1) in the left panel (low C availability), the viruses tend to integrate into the host genome in a lysogenic lifestyle. They harbor AMGs related to C degradation to enhance the cooperative viral‐host survival; 2) in right panel (high C availability), the lytic viruses dominate, leading to host cell lysis and the release of intracellular nutrients. This viral activity triggers two key C pathways. The viral shunt process, where the lysis of host cells releases labile DOC, fuels microbial respiration. While through viral shuttle processes, MBC and MNC stabilizes as MAOC via mineral binding and necromass accumulation. Finally, accelerated OC turnover promotes long‐term SOC sequestration. Arrow thickness indicates process intensity; AMGs, auxiliary metabolic genes; SOC, soil organic carbon; CO_2_, carbon dioxide; O_2_, oxygen; DOC, dissolved organic carbon; MBC, microbial biomass carbon; MNC, microbial necromass carbon; MAOC, mineral‐associated organic carbon.

Emerging studies in aquatic systems demonstrated that viruses regulate C cycling through three interconnected mechanisms: the viral shunt redirects C between particulate and dissolved phases via host lysis, the viral shuttle enhances C export through sticky lysate polymers, and the microbial carbon pump (MCP) stabilizes C through microbial processing—collectively shaping marine and lake C fluxes.^[^ ^37^, ^43^, ^53^ ^]^ At land‐water interfaces, viral impacts intensified with terrestrial organic inputs, primarily through modulating particulate‐dissolved C partitioning during litter decomposition.^[^ ^54^ ^]^ Crucially, these aquatic paradigms found their first terrestrial validation in our soil system, yet with transformative adaptations. Our metagenomic‐microcosm integration revealed soil viruses orchestrate C cycling through dual cooperative mechanisms: host mortality‐driven nutrient turnover and auxiliary metabolic reprogramming (**Figure** **7**).^[^ ^55^ ^]^ Contrasting marine surface‐layer dynamics where lytic shunts dominate, in conditions of low C availability, lysogenic viruses dominated aligned with the strategy of “piggyback‐the‐winner,” where viral shuttle dynamics facilitated host genome integration to enhance mutual survival.^[^ ^37^, ^56^, ^57^ ^]^ These viruses might harbor and express AMGs to supply ATP and degrade organic matter, accelerating the conversion of straw nutrients into bioavailable soil C.^[^ ^58^ ^]^ Conversely, under condition with high C availability, lytic viruses prevailed, triggering host cell lysis and the release of DOC. This “viral shunt” created a metabolic bridge between immediate CO₂ emissions via heterotrophic respiration and long‐term C stabilization through the MCP.^[^ ^42^, ^59^ ^]^ The MCP operated synergistically with viral activities through viral lysis, which enriched microbial necromass precursors and virus‐derived persistent compounds (e.g., capsid proteins) directly contributed to MAOC and MNC formation.^[^ ^43^ ^]^ This tripartite interaction resolved the apparent paradox of viral roles in C cycling, with the viral shunt driving mineralization that releases C in the short term, the viral shuttle enhancing mobility to redistribute substrates, and the MCP enabling long‐term storage. Collectively, these viral activities simultaneously primed short‐term nutrient turnover and architected long‐term C sequestration frameworks.^[^ ^60^ ^]^

## 4 Experimental Section

### 4.1 Soil Sample Collection

The details of the soil samples have been previously described.^[^ ^61^ ^]^ The soil samples were collected from the Fengqiu Agricultural Ecological Experiment Station, Chinese Academy of Sciences, China (35 °01 “N, 114 °32 ”E). This area had a temperate monsoon climate with mean annual rainfall and temperature of 615 mm and 13.9 °C, respectively. The long‐term experimental field was established in 2011 with two methods of straw management and two rates of N application. This resulted in four different treatments, each with three replicates. The treatments are delineated as follows: T1 (straw removal + PK fertilizer, K represents potassium), T2 (straw returning + PK fertilizer), T3 (straw removal + NPK fertilizer), and T4 (straw returning + NPK fertilizer). The rates of fertilizer applied included N fertilizer (210 kg urea ha^−1^ soil), phosphorus fertilizer (157 kg phosphorus pentoxide [P_2_O_5_] ha^−1^ soil), and potassium fertilizer (105 kg potassium oxide [K_2_O] ha^−1^ soil). The physical and chemical properties of the soils are presented in Table S1, Supporting Information. In June 2022 after harvesting the wheat, soil samples (0 to 20 cm depth) were collected by taking five core soils from each plot and mixing them into one composite sample. After collection, all the samples were immediately transported to the laboratory on ice. Each soil sample was divided into two subsamples. One was stored at −80 °C for DNA extraction, and the other was stored at 4 °C to determine the content of nutrients and conduct the microcosm experiments.

The pH value was measured in solution with a 1:2.5 ratio of soil to deionized water (v/v) by a pH meter. The soil organic carbon (SOC) was determined by K_2_Cr_2_O_7_‐H_2_SO_4_ oxidation. The total nitrogen (TN) was measured on a CN analyzer (Vario Max CN, Elementar, Hanau, Germany). Ammonium (NH_4_^+^) and nitrate (NO_3_^−^) were extracted with 2‐M potassium chloride in a 1:10 soil‐solution ratio and determined by a continuous‐flow autoanalyzer (San++ System, Skalar Analytical BV, Breda, the Netherlands). Soil total phosphorus (TP) was determined by the molybdenum blue method through measuring the absorbance at 700 nm on a Shimadzu UV‐1780 UV–vis Spectrophotometer (Shimadzu Corporation, Kyoto, Japan). Available phosphorus (AP) was bicarbonate (NaHCO_3_) at the pH of 8.5 and determined with the same method as described in TP measurement. The contents of MAOC and POC were determined by using a 0.5% solution of sodium hexametaphosphate to extract the C, cause it to disperse, shake, sieve, and dry it, and measure the content of organic C. The content of exchangeable cation Ca^2+^ (Ca_exe_), oxalate‐extractable Fe (Feo), dithionite‐extractable Fe (Fed) and their associated OC components were analyzed using different extraction methods, including ammonium acetate, citrate‐bicarbonate‐dithionite (CBD) and oxalate, respectively.^[^ ^62^, ^63^ ^]^ Furthermore, the DOC was extracted using a 0.5 M solution of potassium sulfate (K_2_SO_4_). The soils were fumigated with chloroform before extraction to measure the MBC. The extracted DOC and MBC were examined using a total organic carbon analyzer (Multi N/C 3100; Analytical Jena, Jena, Germany). The absorbance of DOC at 254 nm was measured using a Shimadzu UV‐1780 UV–vis Spectrophotometer (Shimadzu Corporation, Kyoto, Japan) to calculate the SUVA_254_, which helped to estimate the aromaticity and potential reactivity of the dissolved organic matter.^[^ ^64^ ^]^ The amino sugar components in the soil (MurN, GlcN, and GalN) were identified through hydrolysis and the purification of air‐dried soil samples and subsequent analysis and quantification using high‐performance liquid chromatography (HPLC). Last, the MNC was calculated as previously described.^[^ ^26^ ^]^

### 4.2 Bioinformatic Analysis: DNA Extraction and Sequencing

The viral DNA extraction protocol commenced with rigorous purification of viral‐like particles (VLPs) through sequential physical and enzymatic treatments. Initially, soil samples (1 g) were homogenized with 5 volumes of pre‐cooled Stabilization Buffer (SB, main compositions: 0.2 mol L^−1^ NaCl, 50 mmol L^−1^ Tris‐HCl, 5 mmol L^−1^ CaCl_2_, 5 mmol L^−1^ MgCl_2_; pH 7.5) using mechanical grinding by Magigene Co., Ltd., followed by three cycles of liquid nitrogen freezing‐thawing to lyse viral capsids. After removing particulate matter through centrifugation (12 000 × g, 5 min), the supernatant underwent dual filtration processes: first through a Millex‐HV 0.22 µm filter to eliminate residual bacterial cells and organelles, then via sucrose‐cushioned ultracentrifugation (160 000 × g, 2 h, 4 °C) to concentrate intact VLPs. The resulting pellet was resuspended in 200 µL of SB buffer and treated with DNase I (1 U µL^−1^, TaKaRa Recombinant DNase I, TaKaRa, Dalian, China) at 37 °C for 60 min to degrade extracellular DNA, with enzymatic inactivation achieved by heating (65–75 °C, 10 min) after adding 2 µL Stop Solution (SS). The sample was centrifuged at 2000 rpm for 5 min, and then 200 µL of supernatant was collected and stored at −20 °C. Subsequently, the total DNA and viral DNA were extracted and amplified using a Fast DNA Spin Kit for Soil kit (MP Bio Biomedicals, Santa Ana, CA, USA), a MagPure Viral DNA/RNA Mini LQ kit (Angen Biotech Co., Guangzhou, China), and REPLIg Cell WGA & WTA kit (Qiagen, Hilden, Germany). The quality of DNA amplification products was assessed using 1% agarose gel electrophoresis, a Qubit 3.0 (Thermo Fisher Scientific, Waltham, MA, USA), and a NanoDrop ND2000 spectrophotometer (Thermo Fisher Scientific). Paired‐end sequencing was performed on an Illumina NovaSeq 6000 platform (Illumina, San Diego, CA, USA). The raw sequence data were deposited in the NCBI Sequence Read Archive (SRA), with the accession number “PRJNA1205416” and “PRJNA1205418.”

### 4.3 Quality Control, Assembly, and Identification

The low quality reads in the sequencing data were removed or trimmed using Trimmomatic (v. 0.36) with the default parameters.^[^ ^65^ ^]^ The high quality reads were then utilized for sequence assembly via MEGAHIT (v. 1.1.2).^[^ ^66^ ^]^ The viral contigs were identified using CheckV (v1.5)^[^ ^67^ ^]^ and Virsorter2 (v2.1.0).^[^ ^68^ ^]^ Primary detection using VirSorter2 in “virome” mode with relaxed cutoff (score ≥ 0.5) to maximize sensitivity. Then, Quality filtering via CheckV to remove contigs with < 50% completeness, > 10% contamination, or residual host region. To enhance viral contig identification, this work implemented a tripartite filtering strategy: 1) Contigs were retained if they satisfied all three criteria by vHMMs pipeline: ≥ 5 viral protein family (VPF) hits via HMMER (v3.3.2) against the VOGDB (v94); < 20% of genes annotated to KEGG Orthology terms; and the total number of genes covered with Pfams (v 31.0) ≤ 40%. 2) Contigs with VPF counts ≥ Pfam counts and VPF representing ≥ 60% of total genes. 3) Clustering via CD‐HIT (v4.7) at 95% identity to retain contigs > 5 kb.^[^ ^69^ ^]^ Prodigal (v. 2.6.3) was used to predict the genes on the contigs that were obtained.^[^ ^70^ ^]^ The metagenomic contigs were compared and annotated using DIAMOND against the NR database (Version: 2021.11) of NCBI. Functional annotation information was obtained by comparing the non‐redundant genes with the KEGG database^[^ ^71^ ^]^ to screen for proteins with the highest sequence similarity, with an e‐value ≤ 0.001. Final classification using “hallmark gene” module of VirSorter2 to confirm free viral particles. The longest sequence was designated as the representative sequence. BWA‐MEM (v. 0.7.17) was used for sequence abundance calculation by mapping to the clean data. From this mapping, the Reads Per Kilobase of exon model per Million mapped reads (RPKM) values were calculated from the bam files and used to estimate the relative abundance of the viral contigs.

### 4.4 Taxonomy Assignment and Lifestyle Prediction

Based on the Viral Protein Families (IMG/VR) database, VPF‐Class was employed to assign the taxonomy to the contigs. The final taxonomic classification was determined by comparing the results from VPF‐Class and CheckV, along with information on the method to identify the virus, confidence, and integrity. The lysogenic viruses were identified using two methods. First, lysogenic marker proteins, such as integrate, transposase, invertase, and combine proteins, were downloaded from the Pfam database. The “hmmscan” tool was then used for recognition and hand detection.^[^ ^72^ ^]^ Second, PhaTYP (https://phage.ee.cityu.edu.hk/phatyp) was used as a supplementary tool for prediction.^[^ ^73^ ^]^ The remaining virus contigs were considered potential lytic viruses.

### 4.5 Host Prediction and Function Annotation

Potential hosts of the viruses were predicted with CRISPR match, tRNA match, and genomic homology match methods: 1) CRISPR match: CRISPRCasFinder with default parameters was used to determine and extract the interval sequences from the host reference database.^[^ ^74^ ^]^ BLAST 2.13.0+ was then utilized to perform a spacer query on the virus contigs with parameters “e‐value ≤ 1e^−10^, identity ≥ 95%, and a maximum of two single nucleotide polymers (SNPs);” 2) tRNA match method: tRNAscan‐SE (v. 2.0.9) (July 2021) was employed to identify the tRNAs in the contigs. The tRNAs that were identified were then queried in the host database using the parameters “e‐value ≤ 1e‐10, identity ≥ 95%”;^[^ ^75^ ^]^ 3) Genomic homology match method: A homologous comparison was performed by comparing the virus contigs with the host database. The parameters used were “identity ≥ 70%, query coverage ≥ 75%, e‐value ≤ 1e^−3^, bit score ≥ 50, and hits ≥ 2500 bp.”^[^ ^72^ ^]^ The combination of the DRAMV (v. 1.2.0, auxiliary score: 1 to 3, amg flags: – M and – F)^[^ ^76^ ^]^ and VIBRANT (v1.2.0, defaults parameters) tools was used for virus‐encoded AMG identification.^[^ ^77^ ^]^ Additionally, Phyre2 (http://www.sbg.bio.ic.ac.uk/phyre2) was utilized to predict the structure of the proteins encoded by the AMGs. Finally, the BPROM server (http://www.softberry.com/) and FindTerm server (http://www.softberry.com/) were used separately to predict the promoter and terminator regions of the AMGs.

### 4.6 Microcosmic Experiment

As described by Albright et al. (2022) and Braga et al. (2020), 100 g of each soil sample was mixed with 20 mL of sterile water in a container and incubated overnight at 37 °C.^[^ ^3^, ^4^ ^]^ The viruses were extracted using a 1% potassium citrate buffer (pH 7), followed by ultrasonication (47 kHz, 3 min) on ice with 30 s of manual shaking at every minute, centrifugation (7000 rpm, 4 °C, 20 min), and filtration of the supernatant through a 0.22 µm PVDF syringe Millipore filter (MilliporeSigma, Temecula, CA, USA). After filtration, the liquid was passed through a tangential flow filtration (TFF) system equipped with a Vivaflow 50 membrane package (Sartorius AG, Germany, n.d.) to collect 2 mL of concentrated virus. A whole community of microbial inoculum and 1 g of homogenized soil was mixed with sterile water in a 1:50 ratio (w/v) for each soil type. To make the microbial inoculum (50 × soil dilution) this work added 1 g of soil to 9 mL of sterile water, hereafter referred to as the suspension. This work then added 1 mL of the suspension to 4 mL of sterile water. For each treatment, a homogenized inoculum was prepared by mixing the virus concentrate with the whole microbial inoculum at a ratio of 2:1 (v/v). This work placed three 50 g portions of each soil sample in culture flasks and sterilized them using γ‐radiation.

This work designed the addition of virus (VS) and non‐addition of virus (NS) treatments for each soil. In particular, VS was added to 0.5 mL of microbial inoculum and 1 mL of virus concentrate, while NS was added to 0.5 mL of microbial inoculum and 1 mL of sterile water. Each treatment was repeated three times for each sample of soil, which resulted in a total of 24 micro‐treatments (Figure S3, Supporting Information). The microbial inoculum and virus concentrate always originated from the same soil. All the micro‐treatments were incubated at 25 °C for 35 days. The concentration of carbon dioxide (CO_2_) was measured using the alkaline absorption method at specific time points on days 1, 3, 5, 7, 14, 21, 28, and 35 during the incubation period. After the incubation period, the DOC, MBC, SUVA_254_, and enzyme activity of the soil were measured. The microbial metabolic efficiency, represented by metabolic quotient (qCO_2_), indicates the amount of microbial respiration per unit biomass and was quantified as mg CO_2_‐C mg^−1^ MBC d^−1^.^[^ ^8^ ^]^ The activities of β‐glucosidase (BG) and N‐acetyl‐glucosidase (NAG) were measured using the porous plate fluorescence analytical method. The soil suspension was added to a 96‐well plate that contained the substrate and buffer and incubated at 28 °C for 30 min. This was followed by centrifugation. The fluorescence intensity of the samples was measured using a multifunctional enzyme‐linked immunosorbent assay (Omega BioTek, Norcross, GA, USA) at the excitation and emission wavelengths of 360 and 450 nm, respectively. Standard curves and blank controls with water instead of soil suspension were prepared for accurate quantification.

### 4.7 Statistical Analysis

All the statistical analyses were performed in Microsoft Excel 2019 (Redmond, WA, USA), SPSS 27.0 (IBM, Inc., Armonk, NY, USA), and R 4.2.2. This work used the “vegan” package in R for alpha‐diversity, non‐metric multidimensional scaling (NMDS) analysis, and multivariate similarity analysis (ANOSIM), Spearman rank correlation with permutation (nperm = 999), and Mantel's correlation analysis. The soil samples were divided into four groups (n = 3), including straw returning, straw removal, nitrogen (N) application, and no N application. The co‐occurrence networks of the viral and bacterial communities were then constructed for straw returning and straw removal treatments. This resulted in four co‐occurrence networks. This work used the “psych” package for the correlation matrix analysis, and selected Spearman correlation coefficients based on their thresholds (| r | > 0.7, [FDR] adjusted *p* < 0.01) in R. The correlation was visualized in a co‐occurrence network using Gephi (v. 0.9.2) with the Fruchterman Reingold algorithm. Analyses of variance (ANOVA) were conducted using the Duncan method for significance testing, and the pairwise analysis used a *t*‐test (*p* < 0.05) in SPSS 27.0. Except for the network figures, which were completed in Gephi, all the others were drawn using R and an online plotting tool called chiplot (https://www.chiplot.online).

## 5 Conclusions

This study emphasizes that viral lysis and virus‐host interactions are pivotal drivers of carbon (C) cycling in typical fluvo‐aquic soils. In soils with high C availability, lytic viruses dominate, triggering top‐down regulation via host lysis. This reshapes microbial communities and fuels the microbial carbon pump (MCP), enhancing stable C accumulation as microbial necromass (MNC) and mineral‐associated organic carbon (MAOC). Alternatively, viral auxiliary metabolic genes (AMGs) linked to C fixation amplify microbial metabolism, further promoting soil organic carbon (SOC) sequestration. In the soils with low C availability, a higher abundance of lysogenic viruses prevails, fostering cooperative virus‐host relationships. The AMGs related to C degradation via lysogeny optimize microbial metabolic efficiency, sustaining community survival under nutrient‐limited conditions. In conclusion, by modulating viral activity, we can strategically enhance SOC storage and soil fertility, which is a promising avenue for sustainable agriculture.

## Conflict of Interest

The authors declare no conflict of interest.

## Supporting information



Supporting Information

Supplemental Table 1

## Data Availability

The data that support the findings of this study are available in the supplementary material of this article.
