# Supplementary material for: Impact of Virus‐Mediated Modifications in Bacterial Communities on the Accumulation of Soil Organic Carbon
Source: Adv Sci (Weinh). 2025 May 23;12(30):e06449. doi: 10.1002/advs.202506449 (PMC12376549; doi:10.1002/advs.202506449)
Supplement: Supplementary file 1 — Supporting Information [file ADVS-12-e06449-s002.docx]

**Impact of Virus-Mediated Modifications in Bacterial Communities on the Accumulation of Soil Organic Carbon**

Mingfeng Liu† 1, Guixiang Zhou† 1, Congzhi Zhang 1, Lin Chen 1, Donghao Ma 1, Lijun Zhang 1,4, Chunhua Jia 2, Ling Ma 3, Jiabao Zhang* 1

1 State Key Laboratory of Soil and Sustainable Agriculture, Institute of Soil Science, Chinese Academy of Sciences, Nanjing 211135, China.

2 State Key Laboratory of Nutrient Use and Management, Institute of Agricultural Resources and Environment, Shandong Academy of Agricultural Sciences, Jinan 250100, China

3 College of Land and Environment, Shenyang Agricultural University, Shenyang 110866, China

4 University of Chinese Academy of Sciences, Beijing 100049, China

† These authors contributed equally to this work

*Corresponding author: Prof. Jiabao Zhang; Email: jbzhang@issas.ac.cn

**Supplementary Figures**

Figure S1. Differences in the alpha-diversity of the viral and bacterial communities and potential factors that contribute to the variation in the composition of the viral and bacterial communities.

Figure S2. The composition of viral community with different lifestyles, with viral classification at the family level.

Figure S3. Schematic illustration of the experimental design.

Figure S4. Impact of viral addition on enzyme activity, metabolic quotient (qCO_2_), and predictor importance for qCO_2_.

Figure S5. The co-occurrence networks of the viruses and hosts under low and high C availability treatments.


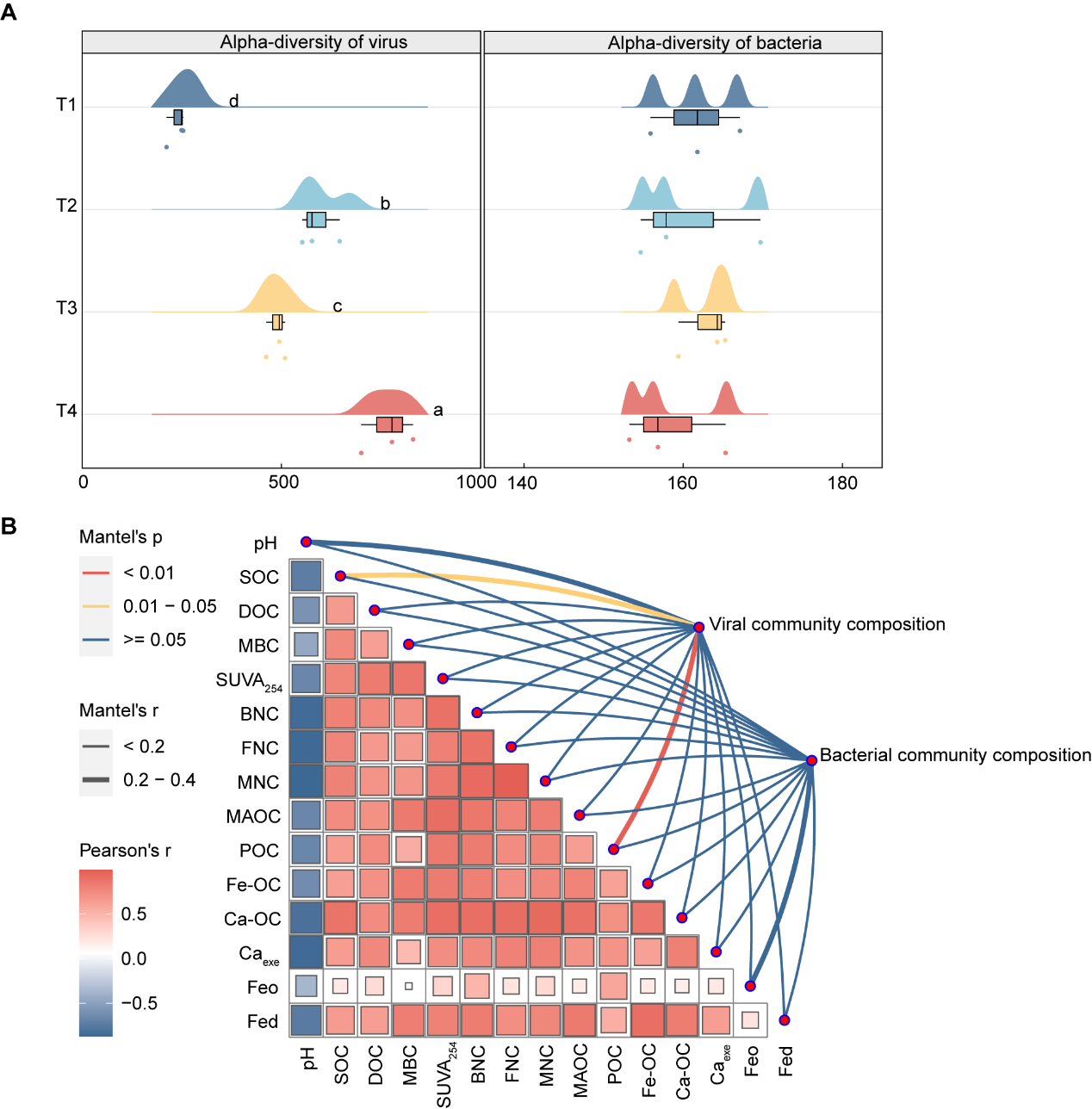


Figure S1. Differences in the alpha-diversity of the viral and bacterial communities and potential factors that contribute to the variation in the composition of the viral and bacterial communities. (A) Alpha-diversity of the viral and bacterial communities from four treatments with significant differences denoted by lowercase letters (ANOVA, *P* < 0.05). (B) The Mantel test between the alpha-diversity of the viral and bacterial communities and SOC components. SOC, soil organic carbon; DOC, dissolved organic carbon; MBC, microbial biomass carbon; SUVA_254_, a typical DOC aromaticity index; BNC, bacterial necromass carbon; FNC, fungal necromass carbon; MNC, microbial necromass carbon; MAOC, mineral-associated organic carbon; POC, particulate organic carbon; Fe-OC, C associated with the reactive Fe mineral phases; Ca-OC, C associated with the cations; Ca_exe_, exchangeable Ca; Feo, oxalate-extractable Fe; Fed, dithionite-extractable Fe; T1, straw removal without N fertilization; T2, straw returning without N fertilization; T3, straw removal with N fertilization; T4, straw returning with N fertilization.


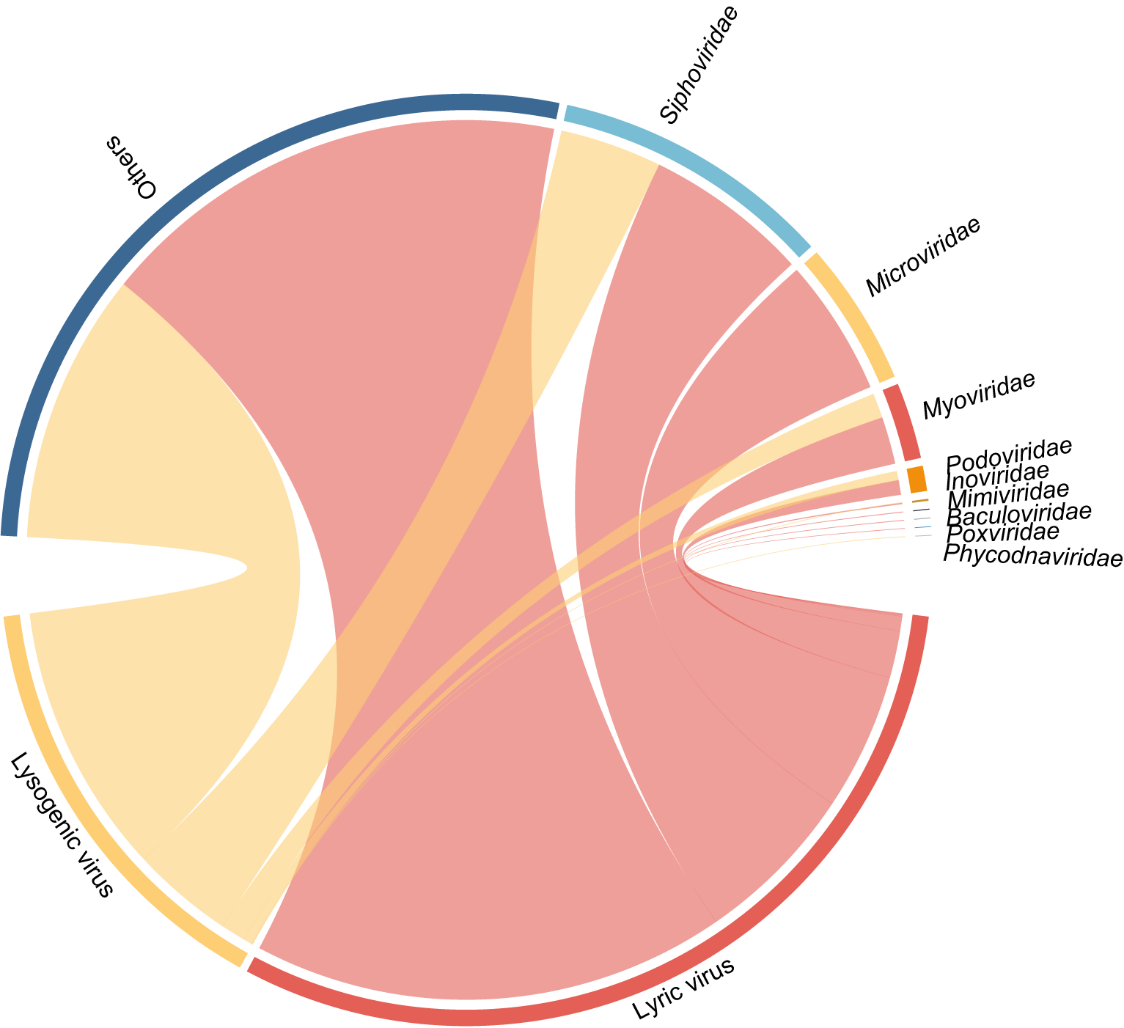


Figure S2. The composition of viral community with different lifestyles, with viral classification at the family level.


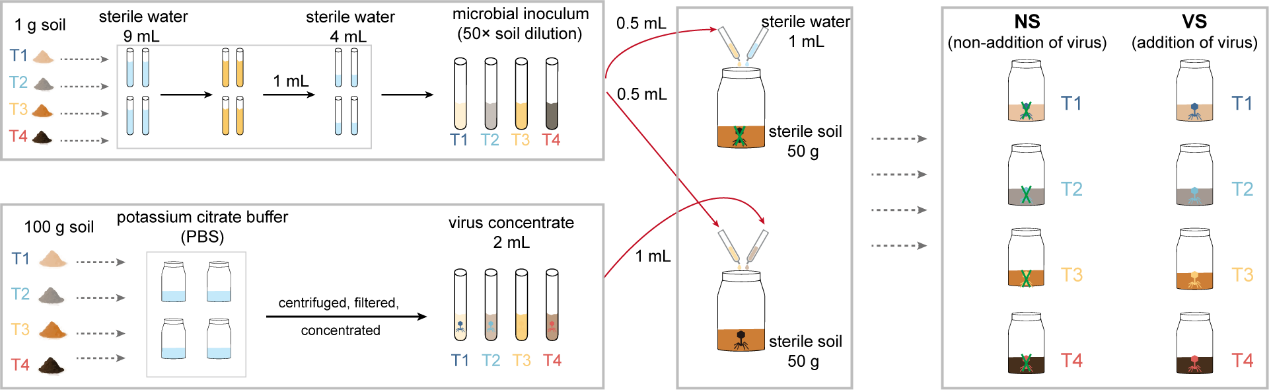


Figure S3. Schematic illustration of the experimental design. Viral fractions from soil suspensions were obtained and concentrated using a tangential flow filtration (TFF) system (see Experimental Section). T1, straw removal without N fertilization; T2, straw returning without N fertilization; T3, straw removal with N fertilization; T4, straw returning with N fertilization.


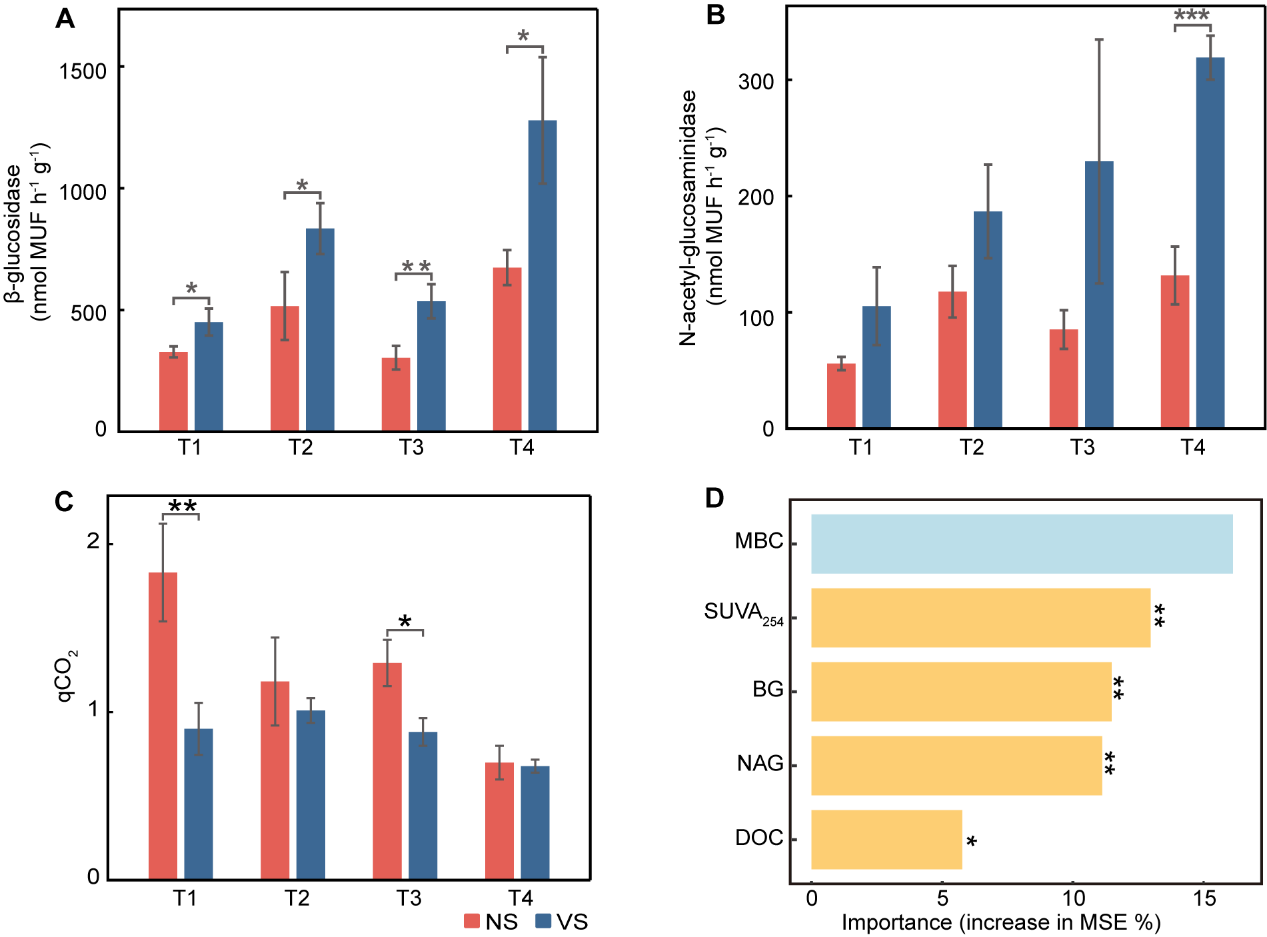


Figure S4. Impact of viral addition on (A, B) enzyme activity, (C) metabolic quotient (qCO_2_, respiration per unit microbial biomass, mg CO_2_–C mg^−1^ MBC d^−1^) and (D) predictor importance for qCO_2_ (percent increased mean square error, MSE), based on a Random Forest analysis. NS, non-addition of virus treatment; VS, addition of virus treatment; T1, straw removal without N fertilization; T2, straw returning without N fertilization; T3, straw removal with N fertilization; T4, straw returning with N fertilization. Significant differences based on a Student’s *t*-test. **P* < 0.05. ***P* < 0.01. ****P* < 0.001.


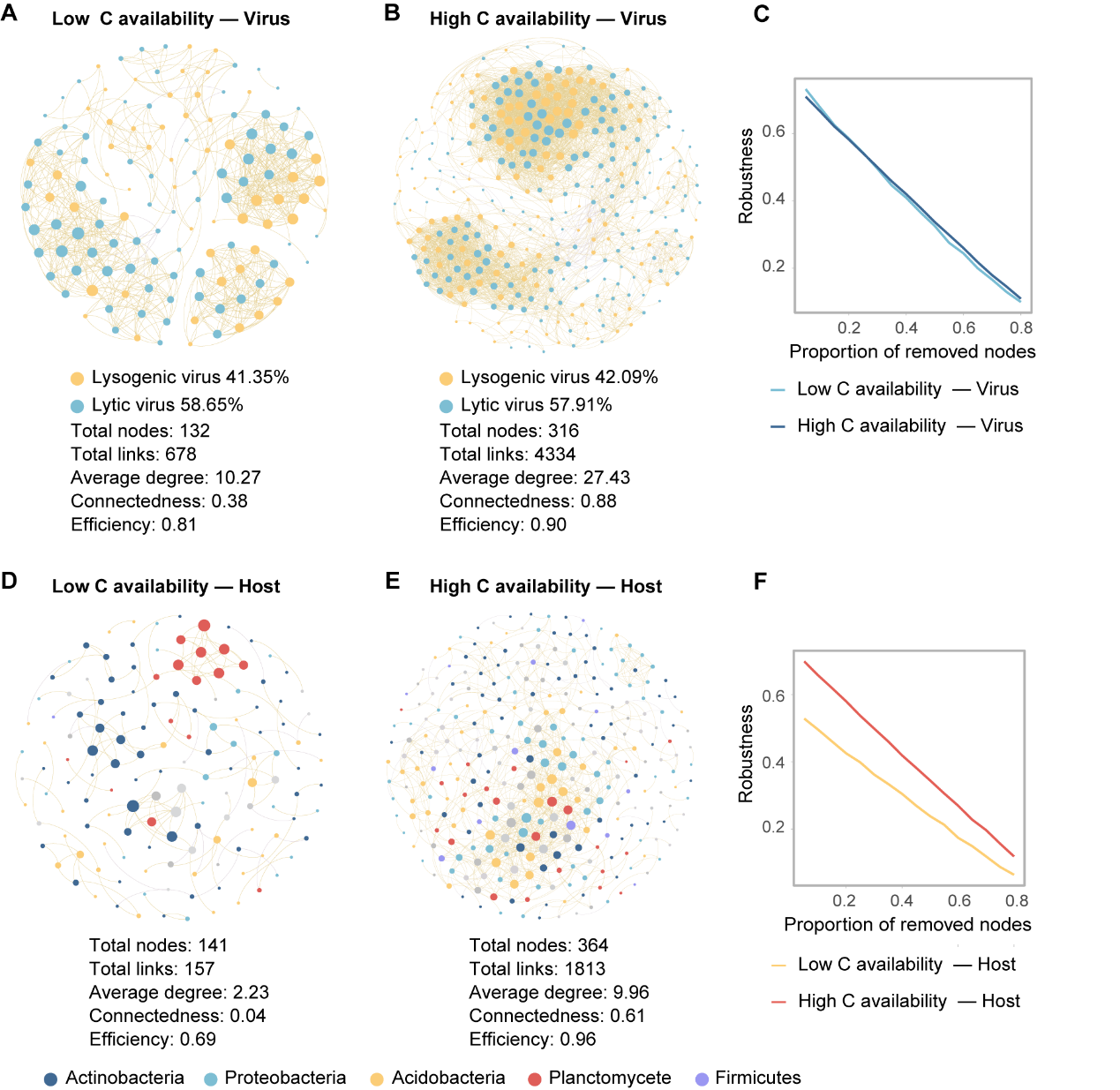


Figure S5. The co-occurrence networks of the viruses and hosts under low and high C availability treatments. Viral networks under low (A) and high (B) C availability treatments. Nodes (circles), viral contigs; yellow nodes, contigs that contain lysogenic virus indicators; blue nodes, lytic viruses; links (lines) between the nodes indicate a correlation, and more links in a node indicate a closer genetic relationship between the contigs. (C) Robustness of viral networks under low and high C availability treatments by randomly removing nodes. Host networks under low (D) and high (E) C availability treatments. Nodes (circles), host contigs, with different colors representing contigs from different phyla; links (lines) between nodes indicate a correlation, and more links in a node indicate a closer genetic relationship between the contigs. (F) Robustness of host networks under low and high C availability treatments by randomly removing nodes.
